# The Impact of Heart Rate Variability Monitoring on Preventing Severe Cardiovascular Events

**DOI:** 10.3390/diagnostics13142382

**Published:** 2023-07-15

**Authors:** Ana-Maria Turcu, Adina Carmen Ilie, Ramona Ștefăniu, Sabinne Marie Țăranu, Ioana Alexandra Sandu, Teodora Alexa-Stratulat, Anca Iuliana Pîslaru, Ioana Dana Alexa

**Affiliations:** 1 Department of Medical Specialties II, Grigore T Popa University of Medicine and Pharmacy, 700115 Iasi, Romania; 2 Department of Medical Oncology-Radiotherapy, Faculty of Medicine, Grigore T Popa University of Medicine and Pharmacy, 700115 Iasi, Romania

**Keywords:** heart rate variability, wearable devices, novel wearable

## Abstract

The increase in the incidence of cardiovascular diseases worldwide raises concerns about the urgent need to increase definite measures for the self-determination of different parameters, especially those defining cardiac function. Heart rate variability (HRV) is a non-invasive method used to evaluate autonomic nervous system modulation on the cardiac sinus node, thus describing the oscillations between consecutive electrocardiogram R-R intervals. These fluctuations are undetectable except when using specialized devices, with ECG Holter monitoring considered the gold standard. HRV is considered an independent biomarker for measuring cardiovascular risk and for screening the occurrence of both acute and chronic heart diseases. Also, it can be an important predictive factor of frailty or neurocognitive disorders, like anxiety and depression. An increased HRV is correlated with rest, exercise, and good recovery, while a decreased HRV is an effect of stress or illness. Until now, ECG Holter monitoring has been considered the gold standard for determining HRV, but the recent decade has led to an accelerated development of technology using numerous devices that were created specifically for the pre-hospital self-monitoring of health statuses. The new generation of devices is based on the use of photoplethysmography, which involves the determination of blood changes at the level of blood vessels. These devices provide additional information about heart rate (HR), blood pressure (BP), peripheral oxygen saturation (SpO^2^), step counting, physical activity, and sleep monitoring. The most common devices that have this technique are smartwatches (used on a large scale) and chest strap monitors. Therefore, the use of technology and the self-monitoring of heart rate and heart rate variability can be an important first step in screening cardiovascular pathology and reducing the pressure on medical services in a hospital. The use of telemedicine can be an alternative, especially among elderly patients who are associated with walking disorders, frailty, or neurocognitive disorders.

## 1. Introduction

Cardiovascular diseases have a real interest worldwide due to their increased incidence. With advancing age, there is an increase in the risk of cardiovascular pathologies, and according to World Health Organization (WHO) statistics, one-third of these deaths occur in people under 70 years old.

Therefore, the only weapon that can identify this risk and reduce the incidence of cardiovascular diseases is prevention. Data from the literature show that heart rate variability (HRV) monitoring represents the variation between two successive heartbeats. HRV is not an arrhythmia; HRV determination is the result of calculating the distance between two consecutive heartbeats, and it is performed using standard electrocardiographic monitoring at rest or Holter ECG monitoring. HRV monitoring is characterized by numerous time, frequency, and non-linear parameters, which are presented in detail below. We can say that a patient has high HRV or low HRV after obtaining the data provided by a special software without having a definite reference interval, as we are used to in medicine [1].

HRV is the result of the autonomic nervous system, and it is caused by the successive actions of the parasympathetic nervous system, which causes a decreased heart rate, and the sympathetic nervous system, which results in increased heart rates and blood pressure.

An increased HRV is correlated with rest, exercise, and good recovery, while a decreased HRV is an effect of stress or illness and functional impairment in older adults. An impairment of HRV can be correlated with many pathological conditions, such as abnormal vegetative tonus, paroxysmal supraventricular tachycardia, or sick sinus syndrome. The presence of high-grade heart failure (class III or IV NYHA (New York Heart Association)) is associated with a decrease in HRV compared to class II NYHA, which is a result of the stimulation of sympathetic tone and a decrease in parasympathetic action. It was also proved that HRV analysis is an independent predictive factor of the ejection fraction [2]. HRV monitoring represents a predominant biomarker of the occurrence of ventricular tachycardia and sudden cardiac death [1].

In a study from 2023 carried out on 192 patients, 82 exhibited hypertrophic cardiomyopathy, 22 had a history of sudden cardiac death, and 88 were part of the control group. The study examined the relationship between HRV and HR values both in the long and short term. Yan et al. demonstrated that most HRV values decreased exponentially with an HR increase, especially in patients with a high risk of sudden cardiac death [3].

Also, in a multicentric (USA and Israel) prospective study conducted on 1043 patients with a low to intermediate probability of developing coronary heart disease, an HRV analysis was performed using Holter ECG monitoring for 1 h, followed by effort echocardiography in 612 patients and myocardial perfusion imaging during exercise in 431 patients. Thus, myocardial ischemia was detected in 66 (6.3%) of the patients, while the results based on HRV analysis show that the frequency of myocardial ischemia was significantly higher among patients with low HRV (11%) compared to high HRV (3%) [4].

Currently, many studies support that an increase in heart rate variability in patients with ischemic heart disease undergoing aerobic or stretching exercise programs improves cardiovascular risk and death rates [2].

It has also been proven that HRV has a direct link to the development or evolution of arterial hypertension. Thus, the determination of a low HRV in prehypertensive people predicts the development of overt hypertension, while in already diagnosed patients, it can guide the readjustment of medication [5].

Also, many studies have proven the correlation between HRV and geriatric syndromes, especially cognitive disorders and neurodegenerative diseases. Decreased HRV leads to a decline in cognition and has a negative impact on people with dementia. This category of pathologies is still insufficiently researched. So, an understanding of the size and direction of any correlation between HRV and cognition and behavior can provide many perspectives for designing prevention and treatment strategies for neuropsychiatric symptoms in dementia [6].

Recent studies compared the effectiveness of the administration of antidepressant treatment to a group of subjects diagnosed with depression. The obtained results confirm a change in HRV parameters correlated with the severity of depression symptoms. This result can be a basis used to support the use of HRV parameters in depression [7].

Therefore, studies have shown that HRV monitoring is important for all people, including those that are young and old and active or sedentary. Young active people have the opportunity to follow their level of physical activity, heart rate, and HRV as a health monitoring factor. On the other hand, with regard to elderly patients, the determination of HRV is an important predictive factor in order to identify the risk of presenting cardio-vascular complications, neuro-cognitive disorders, frailty, and even death.

The quantification of HRV is carried out using time, frequency, and non-linear parameters [8]. 

For example, the domain time parameters are characterized by the following:

1. SDNN—standard deviation of intervals;
2. SDANN—standard deviation of the average intervals for each 5 min segment;
3. RMSSD—root mean square of successive interval differences [2].

SDNN represents the most important parameter in the time domain and an SDNN value of <50 ms indicates an increased cardiovascular risk [8].

The parameters in the frequency domain determine the power distribution in four bands, depending on the power, as follows: ultra low, ≤0.003 Hz; very low, 0.0033–0.04 Hz; low, 0.04–0.15; and high, 0.15–0.4 Hz. They are represented by the following:

1. Power: ULF (ultra-low frequency), VLF (very low frequency), LF (low frequency), and HF (high frequency);
2. Absolute power of ultra-low, very low, low-, and high-frequency bands;
3. Peak: ULF, VLF, LF, and HF;
4. Peak frequency of the ultra-low, very low, low-, and high-frequency bands;
5. LF (low frequency)/HF (high frequency)—ratio of low-to-high frequency power [2].

The parameters in the non-linear domain represent the unpredictability of the time series, and they are represented by the following:

1. S—area of the ellipse, which represents total heart rate variability;
2. ApEn and SampEn—approximate and sample entropy: regularity and complexity of a time series;
3. DFA α1 and α2—detrended fluctuation analysis: short- and long-term fluctuations [2].

## 2. Wearable Devices

The need for the continuous determination of heart rates (HRs) and heart rate variability (HRV) began in 1950 when the monitoring of cardiac function was carried out using portable devices that emitted biopotential signals using 50 MHz radio waves. Later, in 1961, Holter ECG monitoring with built-in storage space was used [2,9]. Over time, using technology, there has been a transformation and an adaptation of digital devices used with different degrees of storage. Beginning in 2020, artificial intelligence-type monitoring is frequently used [2,10]. Current studies confirm the need for the continuous identification and determination of the heart’s electrical activity and the heart rate variability in patients associated with an increased cardiovascular risk, without requiring continuous hospitalization [11].

The following are the available technologies:

- ECG.

The determination of the ECG involves an evaluation of the electrical activity of the heart at different moments of the daily routine (including during night time), at rest or post-effort, and during the period of crisis or lull. This is carried out by mounting six precordial electrodes and four electrodes for limb derivations. Thus, this collected information reveals the heart rate and rhythm and identifies possible signs of acute or chronic ischemia or rhythm/conduction disorders. 

ECG standard monitoring allows the identification of HRV parameters in the time and frequency domain. The parameters in the time domain are quantified by determining the intervals between two successive heartbeats (R-R), which is also called the interbeat interval (IBI). After the correct identification of the R-R intervals, there is the application of some frequency filters (low- and high-amplitude filters) that suppress non-physiological signals [12].

Short-term HRV analysis is frequently used in assessing cardiovascular autonomic function. Several studies carried out confirm the presence of a decrease in HF power and/or an increase in LF power in patients who are associated with myocardial infarction or arterial hypertension [13].
Benefits-Non-invasive, faithful determination-Increased sensitivity and specificity-Painless determination-Does not require continuous hospitalizationDisadvantages-It requires medical personnel trained for the determination and interpretation of the electrocardiogram-Permit a short-term determination, which is limited to the moment when the patient is connected to the electrocardiograph

- Holter ECG monitoring

Of all the devices used to date, Holter ECG monitoring represents the gold standard for determining heart rate variability. In addition to this, important data can be collected with respect to rhythm, heart rate, and the identification of possible rhythm disorders, conduction, or ischemic changes. The main advantage is that it can determine the electrical activity of the heart for 24 h both continuously and non-invasively, offering faithful data with high specificity and sensitivity. It involves the connection of electrodes to the anterior chest; the number of electrodes may vary in different devices. These electrodes are connected to a portable, rectangular device of reduced size and weight. The patient’s electrical heart activity monitorization is stored on a microSD, which is downloaded to a computer after 24 h; the computer has a special program that reads the interpretation. Holter ECG monitoring is a routine assessment that is carried out in any age group, but it is particularly carried out in the elderly population both in patients who present themselves for ambulatory assessments or continuous hospitalization [2].

According to the standards for HRV established by the European Society of Cardiology and the North American Society of Pacing and Electrophysiology, Holter ECG monitoring allows the quantification of frequency-type determinations, time, and non-linear parameters. Also, the Task Force recommends either long-term (24 h) or short-term recordings (5 mins) for the standardized measurement of HRV [14]. Many studies show that HRV parameters decrease with age, especially the time parameters. A study carried out on 240 patients shows that RMSSD decreases faster than the standard deviation of all R-R intervals (SDRR). At the same time, another study demonstrates that HRV parameters touch the maximum values in the morning [15].

Therefore, the determined HRV parameters are also influenced by body temperature, circadian variations, or the sleep cycle [8].
Benefits-Non-invasive, faithful determination.-Increased sensitivity and specificity.-Painless determination.-Allows continuous electrocardiographic monitoring.-Does not require continuous hospitalization.-Maintaining physical activity during the determination is encouraged depending on the patient’s status.Disadvantages-It requires a specialized hospital and trained staff and the installation of the Holter device.-The device cannot come into contact with water: take a shower/bath during the port.-Patients with excessive chest hair need to shave.-The use of gel and leucoplast for attaching the electrodes can cause adverse skin reactions.-Presupposes rest only in the supine position.-May cause inconstant insomnia.

- Smartwatch that uses photoplethysmography

Since 2010, cardiac function monitoring could be carried out continuously by the patient using devices that use photoplethysmography. This method is based on changes in blood volume, which are determined in the blood vessels localized on the wrist of the examined hand. The smartwatch-type device uses an infrared sensor for basic measurements and heart rate changes, and the green LED is used during training and breathing sessions. The smartwatch presents a method that involves the emission of photons that pass through the skin, and by means of a tachogram, the variable intensity of the reflected photons can be measured [2,16,17,18,19].

Smartwatches are used on a large scale globally, and by 2026, it is expected that 20% of the population will have this kind of self-monitoring device. Smartwatches, depending on the production company, show different degrees of sensitivity. Until now, the Apple Watch, Samsung watch, and FitBit wristband have proven to have the best efficiency following the studies carried out. These devices show a good specificity for the direct detection of the possible episodes of arrhythmia, and the information is confirmed by the data below. It does not present a direct way of detecting HRV, but following the discharge of errors, the parameters are calculated based on the performed electrocardiogram and HR [20].

Compared to the Fitbit and Apple devices that use similar LEDs, previous studies state that the use of medical devices that use photoplethysmography results in better correlations with respect to accuracy when using electrocardiographic measurements [2,21].

A recent prospective study conducted by Theurl et al. in 2023 included 104 patients who had acute coronary syndrome with ST-segment elevation that required percutaneous coronary intervention in the first 24 h from the onset of symptoms, 129 patients with stroke confirmed by computer tomography, and 30 patients from a control group. This study compared the values of analyzed HRV parameters following a standard electrocardiographic recording at rest in comparison with the monitoring using a Garmin-type device that uses photoplethysmography. The results of the study confirm the good precision of the smartwatch-type device with respect to parameters that exhibit a lower frequency in cerebrovascular disease patients and those in the control group [22].

A study published in 2018 supports the fact that in patients who required surgical intervention, the use of photoplethysmography devices could differentiate sinus rhythm from atrial fibrillation [23].

Another recent study from 2023 revealed the necessity and applicability of using smartwatches, even for patients who have had episodes of non-permanent atrial fibrillation. Wasserlauf et al. included 30 patients with a mean age of 65.4 ± 12.2 years old, and they used the Apple Watch in parallel with simultaneous recordings from an insertable cardiac monitor (ICM) or cardiac implanted electronic device (CIED). This study demonstrated that atrial fibrillation was recorded in 11 patients who had ICM or CIED, while 8 patients were diagnosed with the help of the Apple smartwatch for a duration of 6 months. Also, it is good to know that in this study, there were no false-positive data. This study supports the use of the Apple smartwatch in atrial fibrillation detection as a screening method, but it needs improvements with respect to sensitivity [24].

This conclusion is underlined by another study carried out on 78 patients from Amsterdam with atrial fibrillation who wore a device that uses photoplethysmography before and after electrical cardioversion. The sensitivity, specificity, and accuracy of the analysis were maintained above 96% in both devices [25].

However, the analysis of a cross-sectional study conducted on 8,203,261 FitBit device users confirms that HRV decreases with age and that the parasympathetic function decreases faster than the sympathetic function. Thus, it was found that the SDRR and low-frequency power parameters show a significant variation depending on gender compared to RMSSD and high-frequency power [15].

Also, a prospective study carried out on 50 adult men (average age of 29 years old and BMI—23 kg/m^2^) who performed physical activity (running and endurance training) demonstrated that the use of smartwatches presents a moderate to high degree of accuracy in many activity speeds [20].
Benefits-Non-invasive determination.-Painless determination.-Allows continuous monitoring while wearing the watch.-Does not require continuous hospitalization.-Maintaining physical activity during the determination is encouraged depending on the patient’s status.-Allows the determination of several parameters (blood pressure, heart rate, heart rate variability, peripheral oxygen saturation, assessment of sleep quality, and physical activity).-Can be used in activities involving contact with water.-Can be used on a large scale by any patient.-Can locate patients with dementia thanks to the built-in GPS device.-User friendly.-Good price.Disadvantages-An inadequate contact between the device and the skin can provide inadequate determinations [23].-Skin color, humidity, or tattoos can affect the accuracy of monitoring [26].-Underestimation of arrhythmias [27].-Ineffective determinations when patients are associated with arrhythmia [27].-They allow the identification of the electrocardiogram in a single derivation [28].

- Chest patch monitors

An alternative to the above-mentioned monitoring is the determination of a patch surface on the patient’s chest, and this is a preferred method compared to Holter ECG monitoring. It is less used today, and it is not routinely recommended for free wear by patients because it requires a recommendation provided by specialized medical personnel. This monitoring method can be used for monitoring time ranges that are between 48 h and 14 days. At the same time, this technique requires specialized medical personnel in order to analyze the data [2,29].

Many studies carried out until now demonstrate that chest patch monitors show a consistently high accuracy during arrhythmia detection, which is comparable to that of ECG Holter monitors [30]. Also, they are even easier to use, can be worn for up to 14 days continuously, and are preferred by patients. A special type of patch (ZioPatch) has been shown to be superior to short-term ECG Holter monitoring in the detection of atrial fibrillation in patients after an ischemic stroke or transient ischemic attack [31].

A study carried out on 74 patients who simultaneously wore chest patch monitors and Holter ECG monitors demonstrated that, in addition to the end of Holter ECG monitoring, 58.1% of patients were identified to have atrial fibrillation, pauses, or sinus tachycardia after 24 h [30].

Also, a study conducted among women who perform occupational and recreational activities demonstrated that the HRV parameters in the time domain (average RR) and frequency domain (HF, LF, and LF:HF) extracted from chest patch monitoring (Actiheart) show similar data compared to three-lead Holter ECG monitoring [14].

Another chest patch device (Firstbeat Bodyguard 2) has been shown to detect 99.95% HR and HRV compared to two-channel Holter ECG monitoring in patients who walked fast on a straight or downhill road or when cycling [14].

Thus, chest-patch-type determinations show values that are comparable to gold-standard determinations [32].
Benefits-Non-invasive determination.-Painless determination.-Allows continuous monitoring during monitoring wear.-Does not require continuous hospitalization.-It is recommended for athletic patients who participate in physical activities.-Can be used on a large scale by any patient.-User friendly.-Being located next to the heart on the surface of the skin, it allows good electrocardiographic monitoring.Disadvantages-Requires trained medical personnel for data interpretation.-It is not widely used by patients.-Requires medical recommendation.

- Chest strap monitor

Chest-strap-type monitoring uses the same method based on photoplethysmography and allows the correct determination of the electrocardiogram continuously. Chest straps that use ECG electrodes provide another wireless solution for measuring HRV. It is used routinely by athletic people. It is preferred over the determinations that use photoplethysmography in peripherals (smartwatch) due to the multiple errors that may appear during training [33]. The superiority of this determination is due to the use of a differential capacitive accelerometer, which is superior to other accelerometers because it has low energy consumption, exhibits quick responses to movements and easier precision. Thus, it allows the detection of the body’s position and the movements of the entire body, offering fewer errors compared to the other determinations [2,28].

Also, chest-strap-type monitoring is considered to exhibit comparative values compared with the gold-standard determination—ECG Holter monitoring. Thus, a study carried out showed that during physical activities of low and moderate intensity, these two determinations show similarities, while during physical activities of high intensity, chest-strap-type monitoring surpassed ECG Holter monitoring (74 errors versus 1332 errors) [14].

The SDNN is the "gold standard" measure for the medical stratification of cardiac risks when recorded over 24 h [14].
Benefits-Non-invasive, painless determination.-Allows continuous monitoring during monitoring wear.-Does not require continuous hospitalization.-It is recommended for athletic patients who participate in physical activities.-Allows use on a large scale by any patient.-User friendly.-Being located next to the heart on the surface of the skin, it allows good electrocardiographic monitoring.Disadvantages-Patients with high weight statuses and the elderly show numerous errors.-It shows a better correlation relative to more intense physical exercise.-In 60% of cases, discrepancies were identified regarding HR and HRV [29].-Less aesthetic compared to smartwatches.

- Upper armband monitors

Monitoring using a band located on the patient’s arm is based on the same technique called photoplethysmography. It is composed of three dry electrodes that stick to the left arm, above which a band is fixed. These three electrodes send ECG signals that are transmitted to a recording device. QRS complexes were automatically detected and recorded based on some algorithms.

A study conducted on 14 patients (10 men) who used upper armband and Holter ECG monitoring during 5 min of monitoring at rest showed that five HRV parameters (SDNN, RMSSD, pNN50, LF, and HF) show very high correlations between the two monitoring methods [34].

Also, it is routinely used in adult patients who perform intense physical activities (cycling or athletics) [2,35,36]. A study carried out on 24 patients who performed an exercise protocol (walking on a treadmill and using a bicycle at different degrees of speed) demonstrated that between Holter ECG and upper armband monitoring, a high correlation in HR determination was observed. So, the upper armband can be used as a valid measure to determine the HR during physical activities of moderate and high intensity [35].
Benefits-Non-invasive, painless determination.-Allows continuous monitoring while wearing the device.-Does not require continuous hospitalization.-It is recommended for athletic patients who participate in physical activities.-Allows the determination of several parameters (heart rate, heart rate variability, peripheral oxygen saturation, body temperature, physical activity, and pedometer).-Use on a large scale by any patient.-User friendly.Disadvantages-There are not enough studies with respect to pediatric patients.

- Ring-shaped biosensors

The use of medical sensors has also been implemented in ring-type devices, which represents a more comfortable and continuous alternative to chest belts. The device uses the same technique described previously, photoplethysmography, which measures the reflectivity of the blood along the entire pulse’s length. It allows the quantification of blood variation at the peripheral level and subsequently the determination of arterial stiffness, HR, and HRV [37,38]. At the same time, a study carried out on four patients whose degree of exposure to stress was measured supported the possibility of long-term monitoring using this method [39]. This method is not used in clinical practice, requiring new studies to strengthen the data mentioned above.
Benefits-Non-invasive, painless determination.-Allows continuous monitoring while wearing the ring.-Does not require continuous hospitalization.-Use on a large scale by any patient.-User friendly.-Affordable price.-Small device.-Allows the monitoring of chronic diseases.Disadvantages-Inadequate contact between the device and the skin can provide inadequate determinations.-Skin color, humidity, or tattoos can affect the accuracy of monitoring.-Underestimation and ineffective determinations in the case of strong ambient light and faster movements.-Less precise in acute pathologies.-Less precise than the standard electrocardiographic determination.

- Clothing monitor

The use of artificial intelligence in current times has led to the creation of new methods of health assessment in patients who follow intensive programs of physical activities. Thus, a pilot study published in 2019 draws attention to the use of a sports bra, but there are not enough data to validate the accuracy of the determinations regarding HR and HRV. However, the use of a smart shirt that contains multiple sensors that evaluate HR, HRV, respiratory rate, posture, and physical activity has, in small studies, shown differences between 4 and 10% in terms of heart rate determination with the help of the electrocardiogram. In sports competitions, the degree of heart rate errors falls between 1.3 and 6.2%, and it is correlated with the degree of exposure to effort [2,40,41]. To confirm and improve the data mentioned above, it Is necessary to carry out new control studies.

## 3. Conclusions

Over time, due to the increasing life expectancy, levels of stress, sedentary lifestyle, or obesity, there has been an increase in cardiovascular pathologies. This fact introduces a continuous challenge in the determination of increased cardiovascular risks and promotes increased screening in order to avoid risks of sudden cardiac death [42].

Therefore, this increased risk led to the creation of new devices for monitoring one’s own health parameters, especially heart rate and heart rate variability. 

The use and promotion of telemedicine on a large scale, both among young patients and especially among the elderly, can reduce medical impact and secondary costs [43]. 

It turned out that the elderly are the most expensive age segment. Therefore, the determination of HRV using previous methods is part of the current trend in which the elderly are encouraged to monitor their main functional parameters at home and not in medical services. Thus, it can be a more reliable alternative for patients who have balance disorders, functional problems, or various other types of disabilities that prevent them from attending the recommended periodic evaluations [44].

Of all the devices shown above, smartwatch devices are used on a large scale by the entire population, young or elderly, representing a first step in the self-monitoring of health status. However, the chest-strap-type determination is preferred by athletic people or those who follow an intensive sports program due to the increased accuracy of HR and HRV data [15].

## Data Availability

Not applicable.
